# Analyzing the challenges faced by recently qualified nurses in radiation therapy nursing at Japan’s National University Hospitals: Uncovering the determinants of difficulty

**DOI:** 10.1016/j.apjon.2023.100347

**Published:** 2023-11-19

**Authors:** Yumiko Tsuchihashi, Yuko Matsunari

**Affiliations:** a Kagoshima University Medical and Dental Hospital, Kagoshima, Japan; b School of Health Sciences, Faculty of Medicine, Kagoshima University, Kagoshima, Japan

**Keywords:** Young nurse, Difficult case, Radiation therapy, Japan’s National University Hospital, The necessary support

## Abstract

**Objective:**

Newly graduated nurses embarking on careers in radiation therapy nursing at Japan’s National University Hospitals face a spectrum of challenges, many of which have received limited attention in existing literature. This study aimed to uncover the primary difficulties encountered by these nurses, with a specific focus on their implications for training and systematic education.

**Methods:**

Employing a survey involving nurses from five prestigious medical institutions in Japan, we explored the real-time experiences and challenges within radiation therapy nursing. Our investigation concentrated on adverse events and the requisite knowledge for effective symptom management. The results illuminated a notable divergence in experiences among nurses, with particular challenges emerging in the treatment of head and neck cancers, especially when combined with chemotherapy. The data emphasized the pivotal role of certified nurses in offering support and knowledge transfer in complex cases, underscoring the importance of peer support and consultations.

**Results:**

The findings underscored a significant variance in experiences among nurses, with specific difficulties encountered in the management of head and neck cancers, particularly in conjunction with chemotherapy. The data highlighted the essential role of certified nurses in providing support and knowledge dissemination in challenging scenarios, underscoring the significance of peer support and consultations.

**Conclusions:**

This study provides an overview of the landscape, highlighting the critical role of peer consultation and the need for advancements in the educational framework for radiation therapy nurses.

## Introduction

In 2007, the Japanese government enacted the basic act on cancer control and produced a basic plan for promotion of cancer control. These were designed to ensure the quality and uniformity of cancer health care.^1^ The aim was to reduce cancer mortality by 20% and “alleviate suffering and enhance the quality of life for all cancer patients and their families.” The documents identified the importance of promoting radiotherapy and chemotherapy, medical personnel training, and implementing palliative care from the early stages of treatment.^2^ Recent advancements in cancer radiotherapy have meant that the role of nurses in this field has become more significant. However, nurses are now more likely to encounter a range of challenging situations, such as providing decision-making support for treatment options, managing adverse events in cancer radiotherapy, and facilitating self-care following treatment completion. Hashiguchi^3^ suggested that nurses play a key role in cancer radiotherapy, saying, “To maximize the therapeutic effects of radiation therapy, preventive self-care and prompt responses to adverse events related to radiation therapy should be implemented to prevent treatment interruption.” Kume suggested that radiotherapy is now applicable at all stages of cancer, from the initial treatment to palliative care at the end of life. Nurses' role in providing appropriate care was reflected in statements in the study like, “I want to understand the treatment plan in detail” and “I want to grasp the treatment plan to provide effective patient care.”^4^ The roles of nurses, and the knowledge they require to support cancer radiotherapy, are therefore well defined. However, numerous studies have indicated that nurses interacting with patients in clinical settings often lack the fundamental knowledge to fulfill their roles effectively.^4^ One suggestion is that nurses may be struggling because they are unable to provide nursing care as they expected.^5^ In a previous study, young nurses with two to five years of experience and limited experience of managing challenging cases in cancer radiotherapy were asked about patients' treatment processes, what they found difficult, and what kind of support they needed when they first qualified.^6^ In this study, we carried out an investigative survey and analyzed the factors that affected the experience of recently qualified nurses. We wanted to help these nurses improve their skills as they gain experience or possibly choose to specialize in cancer care. We examined effective approaches to difficult cases by clarifying the support required for nurses and the content necessary in their continuing education. A previous study^6^ carried out a survey in a single facility to explore recently qualified nurses' responses to challenging cases in radiation therapy nursing, outlining the types of cases they find difficult. However, this study had a small sample size. Other studies have not investigated the content and current status of postgraduate education on radiation therapy nursing at individual facilities or in the broader education system, leaving details unclear. It is therefore important to explore the situation to inform educators and managers at facilities employing recently qualified nurses. Our investigative survey therefore explored the content and system of postgraduate education in radiation therapy nursing and the situation at each facility, targeting hospitals that are members of the National University Hospital Directors' Conference. The goal was to clarify the current situation, analyze the relationships between factors that make radiation therapy nursing difficult for recently qualified nurses, and identify the necessary support and education systems.

In light of these challenges, this study aimed to establish the main difficulties encountered by recently qualified nurses embarking on careers in radiation therapy nursing at Japan's National University Hospitals. Focusing on these challenges and their implications for training and systematic education, the research seeks to bridge the gap in the literature and provide a foundational understanding to inform the development of targeted educational programs and support mechanisms.

## Methods

### Research survey I

**Fig. 1:**
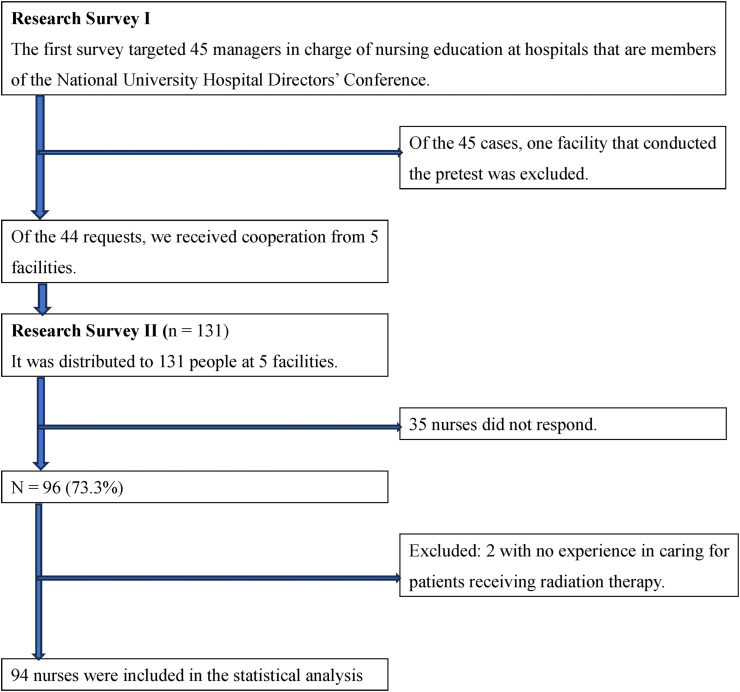
Enrollment of study participants.

The first survey targeted 45 managers in charge of nursing education at hospitals that are members of the National University Hospital Directors’ Conference. It was a fact-finding survey on nursing management using a self-administered questionnaire. The survey period was from September to October 2018. Requests were sent to the 45 target facilities, addressed to the nursing director, asking them to consider agreeing to participate in the survey. If they agreed, the survey was sent to the education manager, who was asked to complete the survey form and mail it back. We also asked the Director of Nursing to consent to collaborate with the second survey, sending their written response back with the completed first survey (see Fig. 1).

The content of the first survey covered the nursing department at the facility. We asked about the number of recently qualified nurses and their highest educational attainment, turnover of nurses in the past year, nursing methodology, work system, and availability of certified and specialized nurses. We also asked about the nursing department's education system including the implementation of the clinical ladder system and new employee training. We then asked specifically about radiation therapy nursing at the facility including whether the facility is a core cancer hospital, the availability of nursing procedures and standards on radiation therapy nursing, training, the availability of pamphlets for patients about treatment, tools for adverse event assessment, and the presence of a consultation system.

### Research survey II

The second survey targeted all nurses in their first to fifth years of service working in hospitals that were members of the National University Hospital Directors’ Conference, and agreed to participate in the research. It used a self-administered questionnaire. The survey was conducted from November 2018 to January 2019.

All nursing departments that agreed to collaborate with the survey were sent questionnaires for all the nurses at the facility. The nursing department was asked to distribute it to target individuals. The participants filled out the questionnaire, and the completed forms were collected by the nursing departments, and then mailed to the research team after the completion deadline. Upon receipt, the research team added the number of the affiliated facility to the package and converted the questionnaire responses to data.

The survey covered basic attributes (gender, age, years of clinical experience, clinical ladder level, department or ward of work, nursing style, preferred source of information to address difficulties at work, and participation in radiotherapy nursing training) and experience of radiation therapy nursing (whether they had been involved in radiation therapy nursing before becoming a nurse, experience in taking care of patients undergoing radiation therapy and at what cancer site, years of nursing experience in radiation therapy nursing, primary nursing experience, materials used during radiation therapy nursing, evaluation of adverse events, and nursing theories used). We then asked about whether the nurses had experience with difficult cases related to radiation therapy nursing and their level of nursing experience at that time including details of the patients involved (treatment site, treatment method, and stage of treatment), other situations that felt challenging, the reasons why the nurses felt challenged, their responses to difficulties and the results of those responses, who provided support in challenging cases, and their thoughts when dealing with challenging situations. Finally, we asked about the knowledge that they considered necessary for patient care during radiotherapy, giving them 15 options with multiple answers possible.

### Ethical considerations

The research was approved by the ethics review committee of Kagoshima University’s School of Medicine and Dental Hospital (IRB No. 170136-Kai-2). All the participants were given a written explanation of the questionnaire survey, and it was administered anonymously. To fully protect the privacy of the participants, we refrained from asking questions that could identify individuals. The data were treated as a group, and no information that could identify individuals or the groups to which they belong was disclosed. Throughout the research period, we ensured the protection of information by securely storing response data and materials, including information about group affiliations, in a locked depository. After the research was completed, all data on the computer were erased, and other materials containing information about group affiliations were shredded and destroyed.

### Definition of terms

In this study, the term “recently qualified nurses” describes nurses who are assumed to have achieved the “competent” and “proficient” stages in Benner's five-stage skill acquisition model of clinical nursing practice and have between 2 and 5 years of nursing experience.

“Radiation therapy” is a blanket term covering three types of therapy: external beam radiation therapy, brachytherapy, and radio isotope (RI) therapy. External beam radiation therapy is a commonly used method that involves irradiating the body from the outside. Brachytherapy is a technique that involves placing a container sealed with a radioactive isotope close to the tumor to irradiate it. Internal radiation therapy (RI therapy) involves the administration of unsealed radioisotopes either orally or through intravenous injection.

## Results

### Research survey I

We obtained agreement to participate in the study from five of the 45 institutions approached. Table 1 summarizes the main characteristics of these five facilities. Of these five institutions, just one had nursing procedures and standards related to radiotherapy nursing. Two facilities offered in-house training in radiotherapy nursing, both delivered by certified cancer radiotherapy nurses. The total training time was 4 hours, covering topics such as the characteristics of cancer radiotherapy, its effects on the human body, and countermeasures for adverse events. Two facilities had assessment tools for adverse events, with evaluations conducted using the Common Terminology Criteria for Adverse Events (CTCAE).^7^ Only two facilities had certified cancer radiotherapy nurses, three had specialist cancer nurses, and one had nurses in training. Three facilities had a consultation system for managing patients undergoing radiotherapy, with the staff in charge consisting of certified cancer radiotherapy nurses, cancer nurses, and palliative care teams. Over the previous year, these three facilities had 5, 10, and 22 consultations, all related to symptom management during radiotherapy.

**Table 1:** Overview of the five participating facilities.

Facility	Last year's nurse turnover rate	Nursing system	Shift pattern	Cancer hub	Clinical ladder system	Certified nurses	Specialist nurses
A	Less than 2%–3%	Team nursing	Mixed	Yes	Implemented	24	3
B	More than 10%	Team nursing	Mixed	Yes	Implemented	18	None, 3 in training
C	Less than 1%	Primary nursing & partnership nursing system	Three shifts	No	Implemented	1	None
D	Less than 1%	Primary nursing	Mixed	No	Implemented	1	1
E	Less than 2%–3%	Partnership nursing system	Two shifts	Yes	Implemented	23	3

### Research survey II

We received 96 responses from nurses at the five participating institutions. Of these, 93 nurses (96.9%) had cared for patients undergoing radiation therapy and were included in the study. The basic attributes of the study participants are summarized in Table 2. There were 7 men and 86 women, with an average age of 25.0 years (standard deviation: 2.8). Their experience of nursing patients receiving radiation therapy is shown in Table 3.

**Table 2:** Basic attributes of the study participants (*N* = 93).

Attributes	With difficult case experience (*n* = 34)	Without difficult case experience (*n* = 59)
Gender		
Male	3	4
Female	31	55
Clinical ladder level		
Level I	22	39
Level II	8	14
Level III	1	1
None	3	5

SD, standard deviation.

**Table 3:** Experience of nursing patients receiving in radiation therapy, by experience of difficult cases (*N* = 93).

Attributes	With difficult case experience (*n* = 34)	Without difficult case experience (*n* = 59)
Experience of people with cancer (as students, or in family)		
Yes	19	32
No	15	27
Experience of nursing patients receiving radiation therapy (years, mean ± SD)	2.9 ± 1.0	2.4 ± 1.1

SD, standard deviation.

Table 4 shows the nurses’ participation in lectures and workshops on radiation therapy. Overall, 39 participants (41.9%) had attended lectures or training on radiation therapy and 54 (58.1%) had not. Of the nurses who had experienced difficult cases, 19 had participated in training programs and 15 had not. Of those without experience with difficult cases, 20 had participated and 39 had not. The nurses mostly reported participating in lectures, with just one combined lecture and exercise session. Overall, 25 of the workshop presenters were certified cancer radiotherapy nurses, three were specialist cancer nurses, and 14 were radiation oncologists (multiple responses were possible).

**Table 4:** Study participants’ participation in lectures and training sessions on radiation therapy, by experience of difficult cases (*N* = 93).

Attributes	With difficult case experience (*n* = 34)	Without difficult case experience (*n* = 59)
Participation in lectures/training sessions		
Yes	19	20
No	15	39
Format of training session		
Lecture only	19	20
Lecture and practice	0	1
Training session instructors (multiple responses possible)		
Certified cancer radiation therapy nurses	14	11
Specialist cancer nurses	3	0
Radiation therapy physicians	5	9

Table 5 shows the responses on handling difficult cases of nursing in radiation therapy. Overall, 34 participants (36.6%) had dealt with difficult cases and 59 (63.4%) had not. The nurses’ average level of experience at the time of handling the difficult case was 1.90 ± 0.78 years. The head and neck region was the most common treatment site for patients in difficult cases. In total, 27 patients received combined radiotherapy and chemotherapy. Most of the patients were in their 70s, and 16 were in the middle stage of treatment. Of the 34 nurses who had experienced difficult cases, 32 had received support (Table 5). Most often, this support came from daily partners, followed by leaders, and then preceptors.

**Table 5:** Attributes of difficult cases experienced by nurses (*N* = 34).

Attributes	Data
Nursing experience at the time of the difficult case (years, mean ± SD)	1.90 ± 0.78
Patient's site of treatment	
Head and neck	15 (44.1%)
Brain	7 (20.6%)
Lung	3 (8.8%)
Uterus	3 (8.8%)
Esophagus	2 (5.9%)
Liver	1 (2.9%)
Rectum	1 (2.9%)
Malignant lymphoma	1 (2.9%)
Bone metastasis	1 (2.9%)
Breast	0
Prostate	0
Patient's treatment	
Radiation therapy only	6
Radiation therapy + chemotherapy	27
Post-surgery radiation therapy	1
Patient's age group	
Children to 30s	0
40s	2
50s	4
60s	8
70s	14
80s and above	6
Patient's treatment phase	
Before treatment	2
Early treatment	3
Radiation therapy + chemotherapy	16
Late treatment	11
After treatment	2
Did you have someone to provide support to you?	
Yes	32 (94.1%)
No	2 (5.9%)
Supporter	
Preceptor	13
Daily partner	19
Leader	18
Assistant head nurse	11
Head nurse	7
Certified nurse	6
Specialized nurse	1
Attending physician	9
Others: pharmacists, peer nurses, senior nurses	3

SD, standard deviation.

We asked the 34 nurses who had handled difficult cases about the knowledge necessary to manage such cases, allowing for multiple responses. Overall, 29 respondents highlighted the importance of understanding radiation and skin care for radiation dermatitis. More than half of the respondents mentioned oral care for mucositis, pain management, emotional support, nutritional support, radiation sickness and fatigue, and nausea and vomiting (Table 6).

**Table 6:** Knowledge required to handle difficult cases in radiation therapy (*N* = 34)

Knowledge categories	Responses, number (percentage)
Radiation	29 (85.3%)
Skincare for radiation-induced dermatitis	29 (85.3%)
Oral care for mucositis	22 (64.7%)
Pain control	21 (61.8%)
Psychological support	20 (58.8%)
Nutritional support	19 (55.9%)
Radiation hangover & fatigue	19 (55.9%)
Nausea & vomiting	17 (50.0%)
Dose distribution	13 (38.2%)
Explanation and guidance to patients and families about exposure	11 (32.4%)
Spiritual pain	11 (32.4%)
Radiation-induced pneumonitis	10 (29.4%)
Bowel & urinary disorders	9 (26.5%)
Support for sexuality	1 (2.9%)

Table 7 shows the comparison between nurses who had experience with difficult cases and those who did not, using a χ^2^ test. The most significant factors were the individuals consulted about work-related problems and their experience as a primary nurse for patients who had undergone radiation therapy. Nurses who had handled difficult cases were more likely to consult certified nurses for advice and, understandably, had more experience handling difficult cases as primary nurses for patients undergoing radiation therapy.

**Table 7:** Comparison of factors between nurses, by experience of difficult cases.

		With difficult case experience, *n* = 34, *n* (%)	Without difficult case experience, *n* = 59 , *n* (%)	Significant
Age, years, mean ± SD		25.0 ± 23.0	24.9 ± 23.0	0.419
Experience as a nurse at the time of the difficult case, years, mean ± SD		2.8 ± 1.1	2.5 ± 1.0	0.310
Experience in the radiological department at the time of the event, years, mean ± SD		2.9 ± 1.0	2.4 ± 1.1	0.065
Who did you consult about your difficulties?	Nurse manager	11 (32.4)	14 (23.7)	0.467
	Deputy nurse manager	13 (38.2)	16 (27.1)	0.353
	Lead nurse manager	25 (73.5)	45 (76.3)	0.807
	Partner nurse	19 (55.9)	26 (44.1)	0.290
	Nursing peer	14 (41.2)	28 (47.5)	0.666
	Preceptor nurse	6 (17.6)	4 (6.8)	1
	Certified nurse	4 (11.8)	0 (0.0)	0.016∗
	Certified nurse specialist	1 (2.9)	0 (0.0)	0.366
	Medical doctor	6 (17.6)	0 (0.0)	0.162
How many times have you provided nursing for patients receiving radiation therapy?*	0	1 (2.9)	1 (1.6)	0.011*
	1	2 (5.8)	11 (18.6)	
	2–3	9 (26.4)	23 (38.9)	
	3–5	10 (29.4)	8 (13.5)	
	5–10	4 (11.7)	13 (22.0)	
	More than 10	8 (23.5)	3 (5.1)	
Do you have any learning materials that you use in your practice of nursing in radiation therapy?	Yes	29 (85.3)	39 (66.1)	0.054
Do you encounter adverse events in your daily nursing practice?	Yes	28 (82.4)	50 (84.7)	0.776

SD, = standard deviation.

* With difficult case experience, *n* = 32; Without difficult case experience, *n* = 58.∗*P* < 0.05.

### Multiple regression analysis

In the multiple regression analysis, the dependent variable was set to reflect the number of years of experience nurses had at the time they encountered difficult cases. The multiple regression analysis aimed to investigate how the knowledge and skills needed to manage difficult cases are reflected in nurses’ educational programs and continuing education and how this translates into practice in the clinical setting. Before selecting the independent variables for analysis, we confirmed the prerequisites for regression analysis such as linearity, homoscedasticity, the absence of multicollinearity, and the assumption of a normal distribution of data for both the independent and dependent variables. The independent variables for institutional affiliation included designation as a cancer center hospital, availability of in-house training, availability of reference materials, and presence of assessment tools for adverse events. The individual independent variables were the number of people available to consult at work, attendance at lectures and workshops, experience with radiation therapy before becoming a nurse, years of experience in radiation therapy nursing, and experience as a primary nurse for patients undergoing radiation therapy. These variables were analyzed using the forced entry method. Table 8 shows the results of the multiple regression analysis. The coefficient of determination, *R*^2^, was 0.59. The adjusted *R*^2^ was 0.35. These values suggest that this model has significant explanatory power. The four variables “designation as a cancer center hospital,” “availability of in-house training,” “participation in lectures and workshops,” and “number of people available to consult at work” all had statistically significant effects on the dependent variable.

**Table 8:** Multiple regression analysis

Variables	*β*	*t*	*P*-value	Significant
Cancer base hospital	0.244	2.142	0.035	∗
Presence of in-hospital training	−0.303	−2.654	0.01	∗∗
Participation in lectures and workshops	0.266	2.539	0.013	∗
Number of people you can consult at work	0.397	4.349	< 0.001	∗∗
Experience with radiotherapy before becoming a nurse	−0.121	−1.225	0.224	
Years of experience in radiation therapy nursing	0.088	0.84	0.403	
Experience as primary nurse for patients undergoing radiation therapy	0.118	1.123	0.265	
Teaching materials for reference	−0.077	−0.83	0.409	
Evaluate adverse events	0.179	1.848	0.068	
*r*	0.588			
*R*^2^	0.345			
Adjusted *R*^2^	0.275			

∗*P* < 0.05, ∗∗*P* < 0.01.

## Discussion

### Enhancing competence in radiation therapy nursing through institutional support and specialized education

The challenges and exigencies in radiation therapy nursing, as revealed through research surveys I and II and our multiple regression analysis, underscore a multi-dimensional problem with institutional, educational, and clinical practice facets. The synthesis of these findings offers a starting point for understanding the interplay between nurse education, institutional support, and clinical application in the specialized field of radiation therapy nursing in Japan.

#### Institutional engagement and educational structures

The five institutions in this study were all national university hospitals in Japan. Only one of those facilities had nursing procedures and standards for radiation therapy nursing, which may imply that the majority of hospitals do not. This lack of standards may reflect the fact that the Japanese Nursing Association does not recognize a specialty in radiation nursing.^8^ There was also a noticeable deficiency in factors to enhance cancer radiation therapy such as facilities equipped with assessment tools for significant adverse events, CTCAE^7^ evaluation, certified cancer radiation therapy nurses, and specialist oncology nurses.^8^ There may also be a need to improve the consultation system related to the nursing care of radiotherapy patients and symptom management for adverse events during radiotherapy. We believe that the field needs specialized nurses with more advanced knowledge.^9^

The conspicuous absence of nursing procedures and standards in the majority of the surveyed national university hospitals highlights a systemic lacuna in institutional readiness. The regression analysis supports this narrative by showcasing the significant influence of institutional attributes such as being a designated cancer center and the availability of in-house training on nursing preparedness. This intersection suggests a need for systemic changes to incorporate structured protocols and standards.

Another noteworthy variable was the number of people whom nurses could consult at work. This was significant in our regression analysis, potentially highlighting the importance of support and collaborative discussions among health care professionals managing challenging cases. In conclusion, the multiple regression analysis highlighted the challenges nurses face, especially when dealing with patients with cancers of the head and neck region who are receiving radiation therapy combined with chemotherapy. It also underscores the foundational significance of peer support and consultation in this field.^9^

#### Training gaps and the spectrum of experience

Hamaguchi et al. noted that radiation therapy nursing for cancer has yet to be fully established as a specialty and that systematic education in this area is rarely provided in basic nursing education or ongoing education within medical institutions.^3^ Nurses therefore learn to practice cancer radiation therapy nursing through self-directed trial and error.

Nurses' reliance on self-taught methods in the absence of formal education in radiation therapy, as reported in our surveys, corresponds with the regression analysis's emphasis on the significance of in-house training and workshops. This confirms the necessity for specialized training and continuing education programs. These will bolster nurses' ability to handle complex cases and also enhance the overall quality of care provided to patients undergoing radiation therapy.

Radiation is commonly used in the medical field, and nurses frequently come into contact with it, but there are limited opportunities for systematic learning. It is believed that basic knowledge about radiation helps nurses provide a rationale for care to patients.

### Clinical implications of advanced education

The head and neck was the most common treatment site in difficult cases, present in 16 patients (47.0%), followed by the esophagus. This finding is consistent with previous research,^6^ where head and neck cancer and esophageal cancer were most prevalent. The most common treatment for these patients was radiation therapy combined with chemotherapy, which is also consistent with previous findings.^6^ The total radiation dose to the head and neck region is 60–70 Gy, which is higher than for other sites, and treatment combining chemotherapy is becoming standard.^10^ This factor is expected to significantly contribute to adverse events during treatment. As a result, patients often suffer severe pain, and it can sometimes be challenging to continue treatment. Nurses are therefore more likely to perceive these cases as difficult.^11^ The nuanced understanding of the clinical demands, particularly in the treatment of head and neck cancers, resonates with the regression findings that highlight the value of educational programs and peer consultations.

When we asked the 34 individuals who had experienced challenging cases in radiation therapy to state the knowledge necessary to manage these difficult cases, the most common response was skin care for radiation dermatitis, provided by 29 out of 34. The same number also mentioned knowledge about radiation. This result was consistent with a previous study.^6^ This previous study made clear that there is no curative treatment for head and neck cancer; the focus is on treating and managing symptoms.^12^ A key skill is to predict the risk of adverse events based on the dose distribution map created during radiotherapy planning and monitor the skin daily. Patients should be given as much self-care support as possible, with nurses filling in the gaps. The course of the treatment could therefore depend on whether the nurse has the necessary knowledge and skills. The consistency in the need for knowledge in managing adverse events such as radiation dermatitis and mucositis reflects a clear demand for targeted educational interventions. These should be aligned with the practical challenges encountered in the field.

### Limitations

This study has provided an overview of the landscape, highlighting the critical role of peer consultation and the need for advancements in the educational framework for radiation therapy nurses. It also had some limitations. In particular, it had a small sample size, limiting the generalizability of the results. However, it provides a preliminary outline of how recently qualified nurses handle difficult cases in cancer radiotherapy. Future qualitative research through interviews could provide further insights and identify problems. Increasing the sample size would improve the reliability and validity of the research results, offering a clearer understanding of the challenges that nurses face in supporting patients through radiotherapy and potential strategies to address them.

### Strategic directions for future development

The converging evidence from both the surveys and regression analysis points toward strategic imperatives. It is essential to develop specialized training programs, establish clear standards, and bolster institutional resources. The predictive power of our regression model, despite its limitations, provides a quantifiable backbone to the qualitative data from the surveys, reinforcing the call for a systemic overhaul in the educational and institutional frameworks governing radiation therapy nursing.

## Conclusions

Both research surveys I and II and our multiple regression analysis converge on the conclusion that there is a significant need for structured educational programs and institutional support in radiation therapy nursing. The development of specialized training, clearer standards, and enhanced institutional resources are essential to ensure that nurses are equipped to manage the complexities of cancer care, particularly in radiation therapy, where the skillset required is highly specialized and constantly evolving.

## Acknowledgements

We extend our sincere gratitude to all the nurses and nursing managers who participated in this research and survey. We also thank Melissa Leffler, MBA, from Edanz (https://jp.edanz.com/ac) for editing a draft of this manuscript.

## CRediT author statement

The authors collectively contributed to the conception and design of the study. Data collection was undertaken by [Y. Tsuchihashi], who also led the data analysis with support from [Y. Matsunari]. All authors were granted complete access to all the data in the study, with the corresponding author bearing the final responsibility for the decision to submit for publication. The corresponding author affirms that all listed authors fulfill the authorship criteria and that no others meeting the criteria have been omitted.

## Declaration of competing interest

All authors have no conflicts of interest to declare.

## Funding

This research received no external funding.

## Ethics statement

The research was approved by the ethics review committee of Kagoshima University's School of Medicine and Dental Hospital (IRB No.170136-Kai-2). Informed consent was obtained from all individual participants included in the study.

## Data availability statement

The datasets generated and/or analyzed during the current study are not publicly available due to privacy and confidentiality commitments made to the participants at the time of data collection. The data are securely stored in a locked archive, accessible only to the research team. Data access requests can be directed to the corresponding author and will be considered on a case-by-case basis, subject to necessary ethical approvals and agreements to maintain the confidentiality of the data.

## Declaration of Generative AI and AI-assisted technologies in the writing process

No AI tools/services were used during the preparation of this work.
